# Evaluation of Fundus Function in Mature Cataract Patients by Visual Electrophysiology

**DOI:** 10.1155/2023/9065094

**Published:** 2023-10-31

**Authors:** Min Zhang, Min Ji, Mengjia Tan, Ying Yu, Huaijin Guan

**Affiliations:** Eye Institute, Affiliated Hospital of Nantong University, Medical School of Nantong University, Nantong 226001, Jiangsu, China

## Abstract

**Purpose:**

To explore the value of visual electrophysiology in evaluating the fundus function of mature cataract patients.

**Methods:**

124 mature cataract patients (153 eyes) were examined before cataract surgery; the examinations included best corrected visual acuity (BCVA), pattern visual evoked potential (PVEP), full-field electroretinogram (ffERG), and multifocal electroretinogram (mfERG). According to the postoperative fundus conditions, the subjects were divided into two groups: the no fundus disease group and the fundus disease group. Approximately one month after the operation, BCVA was measured, and visual electrophysiology was performed on subjects who had a stable fundus condition and had not received treatment for fundus disease.

**Results:**

One month after cataract surgery, BCVA ≤ 0.3 logMAR was found in 60 eyes (96.8%) without fundus disease and 59 eyes (64.8%) with fundus disease. Compared with the group without fundus disease, the preoperative electrophysiological examination of the group with fundus disease showed that the amplitude of ffERG waves and the amplitude density of the P1 wave in the 2nd to 5th rings of mfERG were decreased (all *P* < 0.05). ffERG and mfERG can be used for differential diagnosis of fundus disease (all *P* < 0.05), while PVEP has no significant diagnostic value for fundus disease (all *P* > 0.05). In the group without fundus disease, the amplitude of the PVEP 15′ P100 wave and the amplitude of dark-adapted (DA) 0.01 b-wave, DA 3.0 a-wave, and DA 10.0 a-wave were negatively correlated with postoperative logMAR BCVA (all *P* < 0.05). In the group with fundus disease, the amplitude of PVEP and ffERG and the amplitude density of mfERG were negatively correlated with postoperative logMAR BCVA (all *P* < 0.05). In the eyes of cortical cataracts, some parameters of PVEP, ffERG, and mfERG were significantly different before and after surgery. In the eyes of nuclear cataracts, some parameters of ffERG and mfERG were significantly different before and after surgery. In the eyes of posterior subcapsular cataracts, some parameters of PVEP and ffERG were significantly different before and after surgery.

**Conclusions:**

ffERG and mfERG can be used to detect fundus disease in mature cataract patients. The preoperative visual electrophysiological examination has high clinical value in predicting postoperative vision of mature cataract patients with fundus disease. Different types of cataracts have different effects on electrophysiological examination results. When interpreting the electrophysiological report, it is necessary to consider the existence of cataracts. This trial is registered with 2019-K068.

## 1. Introduction

Cataract surgery is one of the most frequently performed surgeries in the world. Many patients ask doctors about the recovery of vision after cataract surgery and before cataract surgery and have high expectations for cataract surgery. In fact, if the patient has other problems with the fundus, the improvement of vision after surgery is not particularly ideal. Unfortunately, many patients wait until the lens is completely turbid before coming to the hospital for treatment. Because of the opacity of the lens, many ophthalmic examinations to check the structure and function of the fundus cannot be carried out, and the assessment of the condition of the fundus is more difficult. Failure to identify fundus disease before cataract surgery may make patients have unrealistically high expectations or even make patients think that surgery has caused fundus problems [1]. Therefore, it is an important part of preoperative communication between doctors and patients to evaluate fundus function and explain the prognosis before cataract surgery.

At present, the common examination methods for ocular fundus disease include direct/indirect ophthalmoscopy, ocular B-ultrasound, and optical coherence tomography (OCT). Ophthalmoscope and OCT are easily affected by refractive medium turbidity [2]. Although ocular B-ultrasound is not affected by refractive medium turbidity, its accuracy in diagnosing retinopathy is not high [1, 3]. In recent years, objective visual electrophysiology technology has been widely studied by cataract experts [4, 5].

The visual electrophysiological examination includes a series of noninvasive tests to provide objective indicators of functions related to different positions and cell types of the visual system [6]. Although the related research of visual electrophysiology as a preoperative examination of cataract has been reported, the feasibility evaluation of its application in mature cataract patients is different [7, 8]. Moreover, few studies have associated pattern visual evoked potential (PVEP), full-field electroretinogram (ffERG), and multifocal electroretinogram (mfERG) with fundus conditions and postoperative vision. The purpose of this study is to study the evaluation value of visual electrophysiology on the fundus function of mature cataract patients and provide an objective basis for preoperative doctor-patient communication.

## 2. Materials and Methods

### 2.1. Subjects

This study was performed in line with the principles of the Declaration of Helsinki. Approval was granted by the Ethics Committee of the Affiliated Hospital of Nantong University. Informed consent was obtained from all participating subjects after they were given an explanation of the study.

The inclusion criteria included cataract patients with a Lens Opacities Classification System III grade of  ≥3 for nuclear, cortical, or posterior subcapsular cataracts, and their fundus structure could not be observed. The exclusion criteria included patients whose pupils cannot be fully dispersed (<7 mm); patients with a history of vitreous surgery; patients whose fundus could not be seen clearly after cataract surgery; patients with serious complications during and after the operation; and patients who could not cooperate in completing various examinations.

All subjects underwent routine eye examination before an operation, including visual acuity test, optometry, intraocular pressure assessment, Pentacam three-dimensional anterior segment analysis, inspection of the optical biometric instruments, ultrasonic biomicroscopy, ophthalmology ultrasound A scan, ophthalmology ultrasound B scans, fundus photography, and OCT. According to the International Society for Clinical Electrophysiology of Vision (ISCEV) standard, the visual electrophysiological apparatus (RETI-Port/Scan 21, Roland) was used to carry out the visual electrophysiological examination for each subject, including pattern visual evoked potential (PVEP), full-field electroretinogram (ffERG), and multifocal electroretinogram (mfERG).

After cataract surgery, all subjects underwent fundus examination after mydriasis, including indirect fundus endoscopy, fundus photography, ophthalmology ultrasound B scans, and OCT. According to the postoperative fundus conditions, the subjects were divided into two groups: the no fundus disease group and the fundus disease group. Approximately one month after the operation, the subjects with stable fundus condition and without treatment for fundus disease were subject to visual electrophysiological examination, and the best corrected visual acuity (BCVA) was recorded.

### 2.2. PVEP

The PVEP test was performed in accordance with the ISCEV standards in 2016. [9] A black-and-white checkerboard pattern was reversed to stimulate at a frequency of 2 reversals per second (rps). Two check element sizes were used: 1° and 15′. The field size was 17°, the mean luminance was 50 cd/m^2^, and the contrast of the black-and-white checkerboards was 97%. The pupil of the tested eye was in a natural state, and the subjects with ametropia wore glasses for correction. The active electrode (gold cup electrode) was placed 2 cm above the occipital trochanter, the reference electrode (gold cup electrode) was placed on the forehead, and the ground electrode (gold cup electrode) was placed on the mastoid process. Under normal lighting, the subject sat 1 m away from the display screen for examination, recorded with one eye, and wore a light-tight eye mask on the opposite eye. In order to ensure repeatability, PVEP was scanned 64 times each time and recorded twice.

### 2.3. ffERG

The ffERG test was conducted following the ISCEV standards in 2015 [10]. The pupils were dilated to the maximum, at least 7 mm, with 0.5% tropicamide, and the cornea was anaesthetized with 0.5% proparacaine hydrochloride (ALCAINE). The active electrode (gold foil electrode) was placed at the inferior conjunctival fornix, the reference electrode (gold cup electrode) was placed near each orbital rim, and the ground electrode (gold cup electrode) was placed at the forehead. Stimulations were generated by a Ganzfeld Q450 stimulator. After dark adaptation for more than 20 min, dark-adapted (DA) 0.01 ERG, DA 3.0 ERG, DA 3.0 oscillatory potentials (OPs), and DA 10.0 ERG were detected in the dark room in sequence. Then, light-adapted (LA) 3.0 ERG and LA 30 Hz ERG were performed after 10 min of light adaptation.

### 2.4. mfERG

The mfERG test was performed in accordance with the 2021 ISCEV standards [11]. The pupils were dilated to the maximum, at least 7 mm, with 0.5% tropicamide, and the cornea was anaesthetized with 0.5% proparacaine hydrochloride (ALCAINE). The subjects with ametropia wore glasses for correction. The placement of the electrode was the same as that used for ffERG. The resolution was 61 hexagonal stimulation units. The viewing distance from the subject to the monitor was fixed at 30 cm. The view angle was 27°. Under normal lighting, monocular recording was performed, and the opposite eye was equipped with an opaque eye mask.

### 2.5. Statistical Analysis

Statistical analysis was performed using IBM SPSS Statistics for Windows (version 26.0, IBM Corp). Data are presented as the mean ± standard deviation. Visual acuity data were converted to the logarithm of the minimal angle of resolution to calculate the mean. The results of electrophysiological examination before operation in the group without fundus disease and the group with fundus disease were compared by independent sample *t*-test. The area under the receiver operating characteristic (ROC) curve was used to analyze the sensitivity and specificity of visual electrophysiological examination to distinguish whether there was fundus disease or not. The optimal diagnostic cutoff point was obtained by using the maximum value of the Youden index (YI = sensitivity + specificity − 1). Pearson's correlation analysis was used to explore the correlation between the preoperative electrophysiological parameters and the postsurgical logMAR BCVA values. The results of electrophysiological examination before and after operation were compared by paired sample *t*-test. Significance was accepted at the *P* < 0.05 level.

## 3. Results

### 3.1. Clinical Details of Subjects

A total of 124 cataract patients (153 eyes) aged 42–84 years with an average age of 64.91 ± 9.35 years were recruited. Among them, 61 eyes were age-related cataracts, 57 eyes were diabetes cataracts, 34 eyes were complicated cataracts, and 1 eye was traumatic cataract. Visual acuity prior to cataract surgery ranged between logMAR 4.0 and 0.3. The postsurgical classification of the cataract patients based on the fundus condition within one month after cataract surgery resulted in 62 eyes in the group without fundus disease (aged 48–84 years; average 65.77 ± 8.72) and 91 eyes in the group with fundus disease (aged 42–81 years; average 64.32 ± 9.75). There was no significant difference in age between the two groups (*t* = 0.945, *P* = 0.346). Fundus diseases included 37 eyes of diabetes retinopathy, 30 eyes of high myopia, 8 eyes of retinitis pigmentosa, 5 eyes of macular degeneration, 5 eyes of epiretinal membrane, 3 eyes of old uveitis, 1 eye of central retinal vein occlusion, 1 eye of lamellar hole in the macular region, and 1 eye of amblyopia. Intravitreous drug injection was performed in 12 eyes during cataract surgery, and retinal laser treatment was performed in 4 eyes after cataract surgery. BCVA measurement and visual electrophysiological examination were performed in 29 eyes (46.77%) and 47 eyes (51.65%) of the group without and with fundus disease, respectively, one month after the operation. The vision of all cataract patients improved after surgery. The BCVA of the two groups of patients before and after surgery is shown in Table 1. One month after the operation, BCVA ≤ 0.3 logMAR was found in 60 eyes (96.8%) without fundus disease and 59 eyes (64.8%) with fundus disease.

### 3.2. Comparison of Preoperative Visual Electrophysiology between Groups without and with Fundus Disease

The electrophysiological examination results of the group without fundus disease and the group with fundus disease before cataract surgery were compared by independent sample t-test (Table 2). In the group with fundus disease, the peak time of the PVEP 1° P100 wave was delayed, the amplitude of the ffERG wave was decreased, and the amplitude density of the P1 wave in the 2nd to 5th rings of mfERG was decreased (all *P* < 0.05). However, the peak time and amplitude of the PVEP 1° and 15′ P100 waves, and the amplitude density of the P1 wave in the first ring of mfERG were not significantly different between the two groups (all *P* > 0.05).

### 3.3. ROC Curves as Diagnostic Indicators for Detecting Fundus Disease

**Figure 1:**
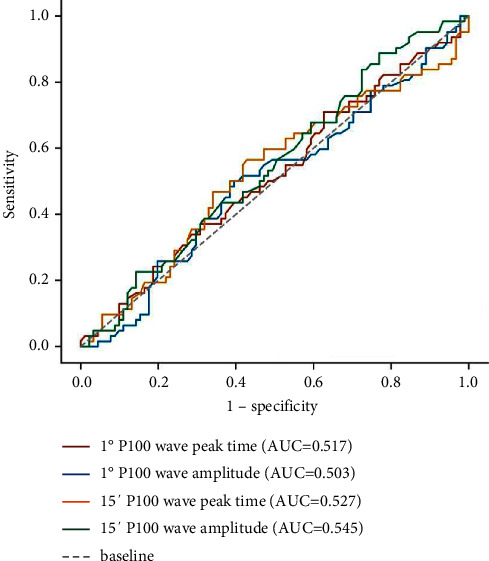
ROC curve of PVEP for detecting fundus disease.

**Figure 2:**
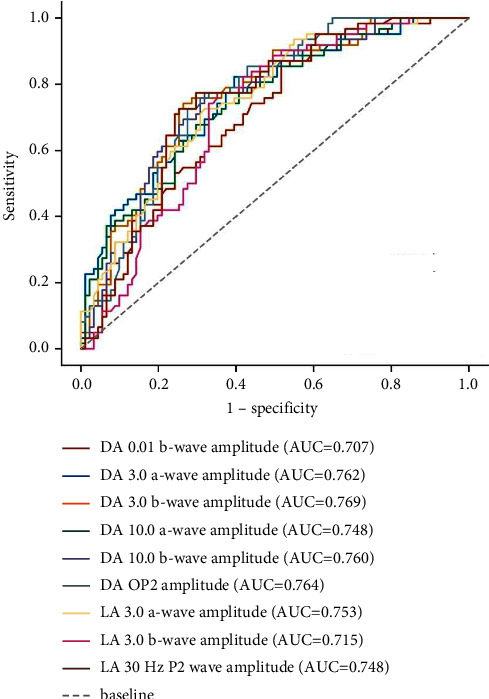
ROC curve of ffERG for detecting fundus disease.

**Figure 3:**
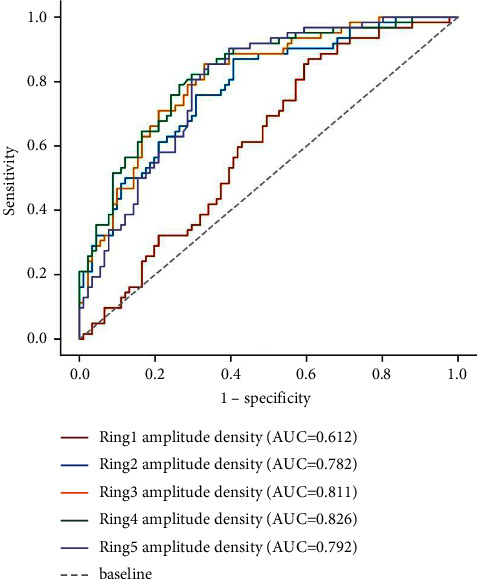
ROC curve of mfERG for detecting fundus disease.

In all cataract patients, the ROC curves of using visual electrophysiology to detect whether there is fundus disease are shown in Figures 1–3. The optimal cut-off value, sensitivity, specificity, and the area under the ROC curve (AUC) of each electrophysiological parameter are shown in Table 3. The amplitudes of each ffERG wave and the amplitude density of P1 waves in the 1st to 5th rings of mfERG can be used to distinguish whether there is fundus disease (all AUC ≥ 0.612, *P* < 0.05), while the peak time and amplitude of PVEP cannot distinguish whether there is fundus disease (all AUC ≥ 0.503, *P* ≥ 0.05).

### 3.4. Correlation between Preoperative Electrophysiological Examination and Postoperative BCVA in the Two Groups

**Figure 4:**
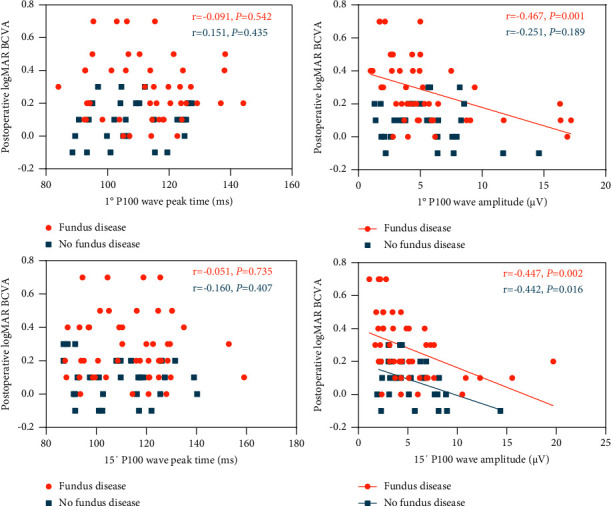
Plots showing the correlation between PVEP parameters and postoperative logMAR BCVA.

**Figure 5:**
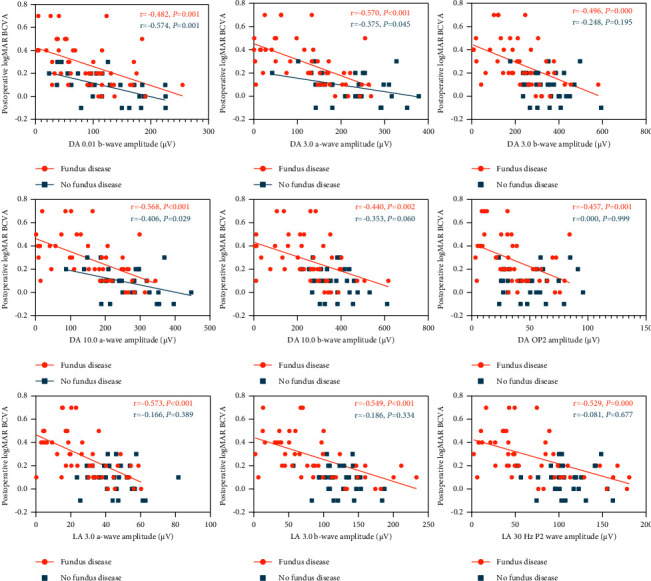
Plots showing the correlation between ffERG parameters and postoperative logMAR BCVA.

**Figure 6:**
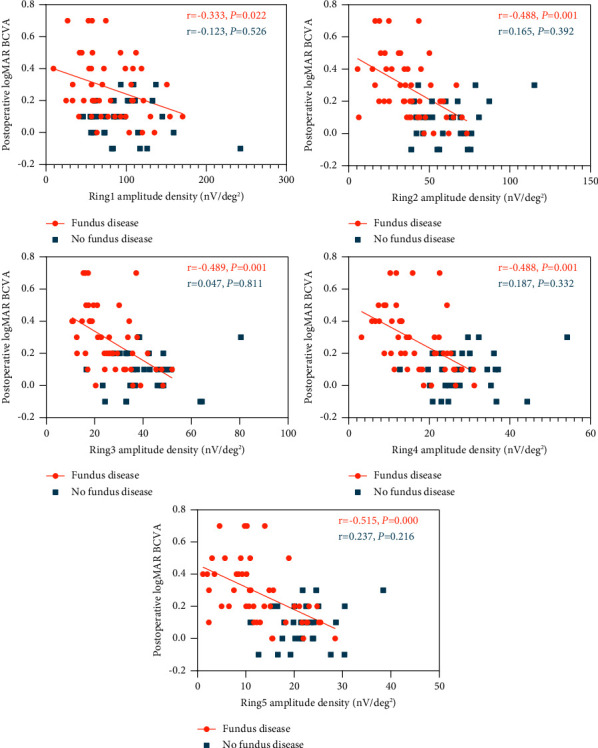
Plots showing the correlation between mfERG parameters and postoperative logMAR BCVA.

In the group without fundus disease, the amplitudes of the PVEP 15′ P100 wave, ffERG DA 0.01 b-wave, DA 3.0 a-wave, and DA 10.0 a-wave were negatively correlated with postoperative logMAR BCVA (all *P* < 0.05) (Figures 4 and 5). The peak time and amplitude of the PVEP 1° P100 wave, the peak time of the PVEP 15′ P100 wave, the amplitude of the ffERG DA 3.0 b-wave, DA 10.0 b-wave, DA OP2 wave, LA 3.0 a-wave, LA 3.0 b-wave, and LA 30 Hz P2 wave, and the amplitude density of the P1 wave in the 1st to 5th rings of mfERG have no significant correlation with logMAR BCVA after the operation (all *P* > 0.05) (Figures 4–6).

In the group with fundus disease, the amplitudes of PVEP and ffERG were negatively correlated with postoperative logMAR BCVA (all *P* > 0.05) (Figures 4 and 5). The amplitude density of the P1 wave in the 1st to 5th rings of mfERG before the operation was negatively correlated with postoperative logMAR BCVA (all *P* > 0.05) (Figure 6). There was no significant correlation between the peak time of the PVEP 1° and 15′ P100 waves and postoperative logMAR BCVA (all *P* > 0.05) (Figure 4).

### 3.5. Changes in Visual Electrophysiology before and after Surgery for Different Types of Cataracts

We classified 76 eyes who underwent postoperative electrophysiological examinations based on the main opacity site of the lens. We analyzed the electrophysiological changes before and after surgery in 26 eyes with cortical cataracts, 27 eyes with nuclear cataracts, and 23 eyes with posterior subcapsular cataracts.

In the eyes of cortical cataracts, after cataract surgery, the amplitude of the PVEP 1° and 15′ P100 waves, the amplitude of the ffERG LA 3.0 b-wave, and the amplitude density of the P1 wave in the 1st, 2nd, and 5th rings of mfERG were higher than before surgery (Table 4).

In the eyes of nuclear cataracts, after cataract surgery, the amplitudes of the ffERG DA 3.0 a-wave, DA 10.0 a-wave, LA 3.0 b-wave, and LA 30 Hz P2 wave, as well as the amplitude density of the P1 wave in the first ring of mfERG, were higher than before surgery (Table 5).

In the eyes of the posterior subcapsular cataract, after cataract surgery, the peak time of the PVEP 15′ P100 wave was delayed, the amplitude of the ffERG DA 0.01 b-wave decreased, and the amplitude of the ffERG LA 3.0 b-wave increased compared to preoperative subcapsular cataract (Table 6).

## 4. Discussion

As early as 1951, researchers used visual electrophysiological techniques to predict the postoperative vision of cataract patients [12]. Subsequent research shows that preoperative visual electrophysiological examination plays an important clinical value in the management of cataract patients with fundus diseases such as diabetes retinopathy, age-related macular degeneration, high myopia, and uveitis [4, 13–15]. In clinical practice, the fundus diseases of cataract patients are complex and diverse. To accurately identify the location of fundus disease, multiple examination methods may be needed. In this study, the combined application of three visual electrophysiological techniques includes the quantitative positioning function of the optic nerve, macular, and each layer of the retina.

PVEP is currently the most important method to detect whether there is conduction dysfunction in optic nerve diseases, and it has the advantages of high sensitivity, stability, and repeatability [6, 16]. Therefore, to detect optic nerve disease before cataract surgery, PVEP was selected for this study. ffERG is the overall response of the retina to a transient flash, which can generally distinguish the dysfunction of the inner or outer retina and the dysfunction of the rod or cone cell system [10, 17]. However, ffERG is mainly produced by the retina rather than the macula, and the role of the macula is very limited. The electrophysiological assessment of macular function requires the use of different techniques, such as pattern ERG or multifocal ERG [6]. mfERG technology is a method to record the local electrophysiological response of the posterior pole retina, which is often used to detect macular diseases. Additionally, mfERG can locate lesions and has a unique diagnostic value for some unexplained diseases that lead to reduced vision but no obvious changes in the fundus, such as cone cell dystrophy, acute regional occult outer retinopathy, occult macular dystrophy, and chloroquine toxic retinopathy [11, 18]. Therefore, to distinguish between macular disease and retinopathy as a whole, we chose mfGER as a supplement to ffERG.

Fundus disease will not only affect the recovery of vision after cataract surgery but also affect the selection of intraocular lenses before surgery and the selection of a surgical plan. For cataract patients with retinal diseases or vision pathway diseases, the results of visual electrophysiology are usually abnormal. Mori et al. [8] found that patients with abnormal TFC results before cataract surgery were usually found to have complications related to retinal or optic nerve damage after surgery. Wang et al. [4] examined the value of standardized electrophysiological techniques in evaluating the retinal function of diabetes cataract patients and found that the DA 10.0 ERG a-wave dominated by rods may give the most sensitive indication of potential diffuse retinal dysfunction and retinopathy. In this study, the amplitude density of each wave of ffERG and the amplitude density of the 2-5th ring of mfERG in cataract patients with fundus disease were significantly lower than those in cataract patients without fundus disease. Both ffERG and mfERG have differential diagnostic values in the diagnosis of fundus disease. Among them, the amplitude density of the fourth ring of mfERG has the highest diagnostic value, with AUC of 0.826, sensitivity of 0.790, specificity of 0.736, and YI of 0.526. In this study, 12 eyes were treated with intravitreal drug injection during cataract surgery, including 10 eyes with diabetes retinopathy, 1 eye with central retinal vein occlusion, and 1 eye with high myopia retinopathy with choroidal neovascularization. The fundus of these 12 eyes could not be seen clearly before an operation, but the OPs of ffERG showed a significant abnormality, suggesting the need for treatment. This shows that visual electrophysiological examination has important clinical guiding significance for the selection of cataract surgery methods.

In our study, almost all cataract patients without fundus disease had a good recovery of vision after surgery, while 35.2% of cataract patients with fundus disease had logMAR BCVA more than 0.3 after surgery. Clinically, we can judge that some cataract patients have fundus lesions according to their medical history, but due to the occlusion of cataracts, we cannot judge the severity of fundus lesions and predict the recovery of visual function after surgery. An et al. [19] performed preoperative electrophysiological examination on 150 cataract patients without obvious other eye diseases and found that the center point amplitude of mfERG, the peak time, and amplitude of ffERG DA 3.0 b-wave were related to postoperative vision, while the peak time and amplitude of PVEP and the amplitude density of mfERG were not significantly correlated with postoperative vision. In this study, we found that the postoperative vision of cataract patients without fundus disease was only correlated with the amplitude of the PVEP 15′ P100 wave, ffERG DA 0.01 b-wave, DA 3.0 a-wave, and DA 10.0 a-wave but not with other indicators. However, we were surprised to find that the amplitude of PVEP, the amplitude of ffERG, and the amplitude density of mfERG were significantly correlated with postoperative vision when we analyzed the correlation between preoperative electrophysiology and postoperative vision in the group with fundus disease. Previously, Ji et al. [14] found that the amplitude and peak time of preoperative ERG were significantly correlated with postoperative BCVA when discussing the visual outcome and prognostic factors of patients with Vogt-Koyanagi-Harada syndrome after cataract surgery. Therefore, we believe that preoperative visual electrophysiological examination has high clinical value in predicting the postoperative vision of cataract patients with fundus disease.

Some researchers believe that mature cataracts itself will not significantly change the results of electrogenesis [7, 20]. Some researchers believe that turbid crystals can reduce the clarity of stimulus pattern contour or contrast of stimulus through defocusing and light-absorption effects, thereby reducing visual electrophysiological parameters [21, 22]. de Waard et al. [23] reported that the different types of cataracts display different light scatter characteristics. Tam et al. [24] reported that the amplitude density of mfERGs was affected differently in the different areas of the retina because of the light scattering by a cataract. In order to investigate the impact of different types of cataracts on electrophysiological examination results, we analyzed the changes in visual electrophysiology before and after surgery for different types of cataracts. Our research results indicate that cortical cataract can affect the results of PVEP, ffERG, and mfERG, with nuclear cataract having minimal impact on PVEP and posterior subcapsular cataract having minimal impact on mfERG. In future clinical work, we need to consider the presence of cataracts when interpreting electrophysiological results reports. In order to better apply electrophysiological examination to cataract patients, we can choose more suitable electrophysiological examination items based on the type of cataract.

In conclusion, the international standard visual electrophysiological examination can be used to detect whether mature cataract patients have fundus disease before surgery and have high clinical value in predicting the postoperative vision of mature cataract patients with fundus disease. Different types of cataracts have different effects on electrophysiological examination results. When interpreting electrophysiological results, we need to consider the presence of cataracts.

## Figures and Tables

**Table 1:** BCVA before and after cataract surgery in two groups.

BCVA (logMAR)	No fundus disease *N* = eyes (%)		Fundus disease *N* = eyes (%)	
	Before surgery	After surgery	Before surgery	After surgery
>1.3	37 (59.68)	0 (0)	40 (43.96)	1 (1.10)
>0.5, ≤1.3	23 (37.10)	0 (0)	44 (48.35)	7 (7.69)
>0.3, ≤0.5	1 (1.61)	2 (3.23)	5 (5.49)	24 (26.37)
≤0.3	1 (1.61)	60 (96.77)	2 (2.20)	59 (64.84)

**Table 2:** Comparison of preoperative visual electrophysiology between groups without and with fundus disease.

	Parameters	No fundus disease (*n* = 62)	Fundus disease (*n* = 91)	*t*	*P*
PVEP	1° P100 wave peak time (ms)	112.49 ± 14.33	111.84 ± 14.51	0.276	0.783
	1° P100 wave amplitude (*µ*V)	5.46 ± 2.90	5.79 ± 3.69	−0.591	0.556
	15′ P100 wave peak time (ms)	114.75 ± 17.53	113.66 ± 16.29	0.391	0.696
	15′ P100 wave amplitude (*µ*V)	5.52 ± 2.94	5.32 ± 3.39	0.372	0.711

ffERG	DA 0.01 b-wave amplitude (*µ*V)	133.89 ± 64.14	88.60 ± 60.15	4.451	<0.001
	DA 3.0 a-wave amplitude (*µ*V)	206.78 ± 75.02	130.60 ± 75.70	6.133	<0.001
	DA 3.0 b-wave amplitude (*µ*V)	346.99 ± 101.84	231.08 ± 123.65	6.103	<0.001
	DA 10.0 a-wave amplitude (*µ*V)	254.58 ± 80.60	170.33 ± 93.19	5.792	<0.001
	DA 10.0 b-wave amplitude (*µ*V)	366.58 ± 98.89	254.24 ± 132.19	5.691	<0.001
	DA OP2 amplitude (*µ*V)	51.36 ± 18.45	32.12 ± 20.03	6.021	<0.001
	LA 3.0 a-wave amplitude (*µ*V)	43.51 ± 15.72	28.48 ± 15.32	5.897	<0.001
	LA 3.0 b-wave amplitude (*µ*V)	119.43 ± 34.96	84.96 ± 51.81	4.914	<0.001
	LA 30 Hz P2 wave amplitude (*µ*V)	104.49 ± 31.97	70.66 ± 42.86	5.585	<0.001

mfERG	Ring1 amplitude density (nV/deg^2^)	91.13 ± 36.21	79.45 ± 42.21	1.778	0.077
	Ring2 amplitude density (nV/deg^2^)	57.22 ± 18.11	38.13 ± 16.63	6.725	<0.001
	Ring3 amplitude density (nV/deg^2^)	40.50 ± 12.22	25.92 ± 11.39	7.548	<0.001
	Ring4 amplitude density (nV/deg^2^)	27.58 ± 8.64	16.98 ± 7.44	8.104	<0.001
	Ring5 amplitude density (nV/deg^2^)	21.00 ± 6.72	13.10 ± 6.99	6.97	<0.001

**Table 3:** Optimal cut-off point, sensitivity, specificity, and AUC of each electrophysiological parameter.

	Parameters	Optimal cut-off	Sensitivity	Specificity	YI	AUC	*P*
PVEP	1° P100 wave peak time (ms)	104.05	0.710	0.374	0.083	0.517	0.714
	1° P100 wave amplitude (*µ*V)	5.43	0.500	0.604	0.104	0.503	0.945
	15′ P100 wave peak time (ms)	116.23	0.565	0.571	0.136	0.527	0.573
	15′ P100 wave amplitude (*µ*V)	2.55	0.887	0.231	0.118	0.545	0.343

ffERG	DA 0.01 b-wave amplitude (*µ*V)	76.80	0.855	0.484	0.338	0.707	<0.001
	DA 3.0 a-wave amplitude (*µ*V)	152.10	0.823	0.604	0.427	0.762	<0.001
	DA 3.0 b-wave amplitude (*µ*V)	292.05	0.742	0.736	0.478	0.769	<0.001
	DA 10.0 a-wave amplitude (*µ*V)	220.85	0.677	0.703	0.381	0.748	<0.001
	DA 10.0 b-wave amplitude (*µ*V)	302.35	0.774	0.681	0.456	0.760	<0.001
	DA OP2 amplitude (*µ*V)	41.20	0.726	0.725	0.451	0.764	<0.001
	LA 3.0 a-wave amplitude (*µ*V)	35.75	0.726	0.681	0.407	0.753	<0.001
	LA 3.0 b-wave amplitude (*µ*V)	99.25	0.774	0.648	0.423	0.715	<0.001
	LA 30 Hz P2 wave amplitude (*µ*V)	89.90	0.774	0.703	0.477	0.748	<0.001

mfERG	Ring1 amplitude density (nV/deg^2^)	58.00	0.871	0.396	0.267	0.612	0.019
	Ring2 amplitude density (nV/deg^2^)	40.43	0.871	0.593	0.464	0.782	<0.001
	Ring3 amplitude density (nV/deg^2^)	30.22	0.855	0.670	0.525	0.811	<0.001
	Ring4 amplitude density (nV/deg^2^)	22.06	0.790	0.736	0.526	0.826	<0.001
	Ring5 amplitude density (nV/deg^2^)	14.86	0.855	0.659	0.514	0.792	<0.001

**Table 4:** Changes in visual electrophysiology before and after cortical cataract surgery.

	Parameters	Before surgery	After surgery	*t*	*P*
PVEP	1° P100 wave peak time (ms)	110.83 ± 11.60	111.25 ± 11.27	−0.145	0.886
	1° P100 wave amplitude (*µ*V)	5.97 ± 4.29	8.35 ± 5.64	−3.529	0.002
	15′ P100 wave peak time (ms)	116.72 ± 15.95	117.82 ± 11.98	−0.34	0.736
	15′ P100 wave amplitude (*µ*V)	5.51 ± 2.45	10.31 ± 8.28	−3.508	0.002

ffERG	DA 0.01 b-wave amplitude (*µ*V)	116.10 ± 53.71	136.47 ± 82.59	−1.189	0.246
	DA 3.0 a-wave amplitude (*µ*V)	185.88 ± 83.16	197.32 ± 87.67	−1.249	0.223
	DA 3.0 b-wave amplitude (*µ*V)	293.92 ± 127.64	320.19 ± 148.30	−1.317	0.200
	DA 10.0 a-wave amplitude (*µ*V)	232.49 ± 93.87	235.80 ± 94.41	−0.352	0.728
	DA 10.0 b-wave amplitude (*µ*V)	316.92 ± 129.77	338.83 ± 146.17	−1.173	0.252
	DA OP2 amplitude (*µ*V)	49.71 ± 25.19	52.07 ± 27.82	−0.675	0.506
	LA 3.0 a-wave amplitude (*µ*V)	39.84 ± 17.06	41.68 ± 17.77	−1.019	0.318
	LA 3.0 b-wave amplitude (*µ*V)	114.50 ± 49.41	135.50 ± 63.45	−3.319	0.003
	LA 30 Hz P2 wave amplitude (*µ*V)	96.07 ± 45.23	101.27 ± 58.19	−0.804	0.429

mfERG	Ring1 amplitude density (nV/deg^2^)	84.13 ± 32.35	124.97 ± 43.08	−4.372	<0.001
	Ring2 amplitude density (nV/deg^2^)	47.11 ± 19.96	59.49 ± 21.83	−3.949	0.001
	Ring3 amplitude density (nV/deg^2^)	33.98 ± 14.33	35.11 ± 15.08	−0.596	0.557
	Ring4 amplitude density (nV/deg^2^)	23.43 ± 9.04	22.58 ± 10.61	0.637	0.530
	Ring5 amplitude density (nV/deg^2^)	17.60 ± 7.46	20.05 ± 9.08	−2.656	0.014

**Table 5:** Changes in visual electrophysiology before and after nuclear cataract surgery.

	Parameters	Before surgery	After surgery	*t*	*P*
PVEP	1° P100 wave peak time (ms)	111.74 ± 14.46	112.87 ± 20.88	−0.22	0.828
	1° P100 wave amplitude (*µ*V)	5.29 ± 3.81	6.01 ± 2.47	−0.979	0.337
	15′ P100 wave peak time (ms)	109.71 ± 17.20	118.86 ± 24.65	−1.406	0.172
	15′ P100 wave amplitude (*µ*V)	5.17 ± 4.29	5.24 ± 3.05	−0.071	0.944

ffERG	DA 0.01 b-wave amplitude (*µ*V)	85.06 ± 53.29	83.13 ± 47.44	0.201	0.842
	DA 3.0 a-wave amplitude (*µ*V)	129.86 ± 70.56	150.07 ± 60.71	−2.432	0.022
	DA 3.0 b-wave amplitude (*µ*V)	246.00 ± 107.59	264.81 ± 115.32	−1.054	0.302
	DA 10.0 a-wave amplitude (*µ*V)	168.89 ± 80.73	187.07 ± 64.57	−2.136	0.042
	DA 10.0 b-wave amplitude (*µ*V)	275.25 ± 107.27	287.07 ± 112.97	−0.713	0.482
	DA OP2 amplitude (*µ*V)	37.14 ± 18.07	35.41 ± 15.73	0.544	0.591
	LA 3.0 a-wave amplitude (*µ*V)	32.14 ± 17.00	34.11 ± 15.49	−0.711	0.483
	LA 3.0 b-wave amplitude (*µ*V)	91.50 ± 53.22	129.61 ± 92.45	−2.309	0.029
	LA 30 Hz P2 wave amplitude (*µ*V)	76.83 ± 41.25	83.63 ± 41.66	−2.242	0.034

mfERG	Ring1 amplitude density (nV/deg^2^)	81.73 ± 32.68	96.36 ± 28.77	−2.377	0.025
	Ring2 amplitude density (nV/deg^2^)	45.68 ± 18.80	49.06 ± 17.31	−1.376	0.180
	Ring3 amplitude density (nV/deg^2^)	30.53 ± 10.81	28.82 ± 10.87	1.059	0.299
	Ring4 amplitude density (nV/deg^2^)	19.67 ± 8.33	20.51 ± 8.00	−0.901	0.376
	Ring5 amplitude density (nV/deg^2^)	15.44 ± 7.37	16.99 ± 7.29	−1.689	0.103

**Table 6:** Changes in visual electrophysiology before and after posterior subcapsular cataract surgery.

	Parameters	Before surgery	After surgery	*t*	*P*
PVEP	1° P100 wave peak time (ms)	109.72 ± 14.99	107.15 ± 11.72	0.706	0.488
	1° P100 wave amplitude (*µ*V)	5.13 ± 3.04	5.99 ± 2.71	−1.283	0.213
	15′ P100 wave peak time (ms)	108.73 ± 14.88	120.61 ± 16.47	−2.284	0.032
	15′ P100 wave amplitude (*µ*V)	4.94 ± 3.04	5.72 ± 3.58	−0.871	0.393

ffERG	DA 0.01 b-wave amplitude (*µ*V)	107.93 ± 72.88	81.72 ± 51.03	2.45	0.023
	DA 3.0 a-wave amplitude (*µ*V)	171.89 ± 115.26	169.75 ± 98.69	0.255	0.801
	DA 3.0 b-wave amplitude (*µ*V)	267.17 ± 156.94	262.35 ± 146.85	0.433	0.669
	DA 10.0 a-wave amplitude (*µ*V)	209.48 ± 129.91	200.00 ± 111.63	0.977	0.339
	DA 10.0 b-wave amplitude (*µ*V)	290.46 ± 169.66	278.38 ± 151.07	0.905	0.375
	DA OP2 amplitude (*µ*V)	36.78 ± 23.03	36.77 ± 20.62	0.004	0.997
	LA 3.0 a-wave amplitude (*µ*V)	31.80 ± 18.05	34.90 ± 21.79	−1.639	0.116
	LA 3.0 b-wave amplitude (*µ*V)	97.92 ± 49.80	114.02 ± 66.99	−2.331	0.029
	LA 30 Hz P2 wave amplitude (*µ*V)	81.53 ± 49.45	87.11 ± 53.84	−1.344	0.193

mfERG	Ring1 amplitude density (nV/deg^2^)	92.72 ± 52.61	103.98 ± 38.43	−0.953	0.351
	Ring2 amplitude density (nV/deg^2^)	48.04 ± 22.84	49.60 ± 19.68	−0.545	0.591
	Ring3 amplitude density (nV/deg^2^)	32.14 ± 15.97	29.01 ± 13.90	1.472	0.155
	Ring4 amplitude density (nV/deg^2^)	20.94 ± 11.40	20.69 ± 8.46	0.155	0.878
	Ring5 amplitude density (nV/deg^2^)	16.28 ± 9.31	17.79 ± 9.65	−1.55	0.135

## Data Availability

The data that support the findings of this study are available from the corresponding author upon reasonable request.
